# Estimated Manipulation of Tablets and Capsules to Meet Dose Requirements for Chinese Children: A Cross-Sectional Study

**DOI:** 10.3389/fped.2021.747499

**Published:** 2021-10-29

**Authors:** Liwen Zhang, Yan Hu, Panpan Pan, Chengtao Hong, Luo Fang

**Affiliations:** ^1^Department of Pharmacy, The Children's Hospital, National Clinical Research Center for Child Health, Zhejiang University School of Medicine, Hangzhou, China; ^2^Department of Pharmacy, The Cancer Hospital of the University of Chinese Academy of Sciences (Zhejiang Cancer Hospital), Institute of Basic Medicine and Cancer (IBMC), Chinese Academy of Sciences, Hangzhou, China

**Keywords:** capsule, manipulation, pediatric, tablet, prescription, drug dose

## Abstract

**Objectives:** To estimate the frequency of manipulations of all tablets and capsules prescribed for children in a teaching and tertiary children's hospital in China over the course of 1 month. Moreover, hypothetical reduction of manipulation according to the availability of low-strength tablets/capsules licensed by the Chinese National Medical Products Administration (CNMPA) was evaluated.

**Methods:** Information on all tablets and capsules prescribed in the hospital from March 17 to April 16, 2019 was collected. It was assumed that tablets or capsules were manipulated if the prescribed dose would have required only a proportion of the intact dose form. Manipulation typically includes splitting or crushing tablets, opening capsules and dispersing in water, or combinations of these method. Moreover, we defined an “avoidable manipulation,” when the dose could be rounded and/or when alternative products with a reduced strength or in liquid formulation were available in the hospital, and a “inappropriate manipulation,” which involved manipulated medications with a direct contraindication for any manipulation, such as those with a narrow therapeutic index or hazardous ingredients, or modified release dosage-forms. The frequencies of total, avoidable, and inappropriate manipulation were estimated, along with the hypothetical reduction of manipulation according to the availability of CNMPA-approved drug doses.

**Results:** A total of 17,123 prescriptions for 142 medications were identified to have required a manipulation among 78,366 prescriptions administered during the study period, with 43 different proportions of subdivisions, ranging from a 19/20 to 1/180 product strength reduction. Half, quarter, and trisection were the most common subdivisions administered. Overall, 19% of the manipulated prescriptions were determined to be avoidable, and 19% of the manipulations involved medications with a clear recommendation to not manipulate. In addition, 21% of the manipulated prescriptions could have been potentially avoided if all of the approved preparations with the lowest strength would have been available at the hospital. Any manipulations undertaken were carried out by pharmacists and family care givers.

**Conclusions:** More than 20% of tablets and capsules prescriptions need manipulated, included a high incidence of avoidable and inappropriate manipulation.

## Introduction

Manipulation of medications is a common practice in clinical settings to achieve the required dose for a given patient, particularly for children owing to the shortage of age-appropriate formulations of many drugs (1–3). Manipulated drug dosages generally have a comparable therapeutic effect to intact prescriptions (4–9). Nevertheless, manipulation of tablets or capsules, or the dosage form, might cause additional adverse events owing to delivering an inaccurate dose for the patient's weight. According to a systematic review by Richey et al. (10), only 20 out of 64 manipulated products met the USP weight specification of subdivision, which could lead to administration of an inaccurate dose. Similarly, a change to the dosage form might influence the pharmacokinetic and pharmacodynamic profile of a drug, especially for sustained-release or enteric-coated tablets. For example, studies have shown that subdivision of tablets resulted in reduced bioavailability (11), a faster time to reach peak concentrations (12, 13), and higher peak plasma concentration levels (14, 15) of the drug compared with those of the intact tablet. One study demonstrated that crushing a pentoxifylline tablet (extended-release, Trental) was associated with a higher risk of nausea, diaphoresis, headache, and vomiting compared with the intact tablet (12). Moreover, the unpleasant taste of the crushed tablets is highly noticeable to children, which in turn could provoke further non-compliance (16). However, the specific risks of such drug dose or form manipulations in children remain largely unknown. Moreover, the impact of such manipulations on the occupational safety of pharmacists, and on the pollution of the water and environment has not been investigated, and could have some serious or unexpected implications.

The extent to which prescriptions are manipulated in the clinical setting largely varies among regions, and is significantly associated with the availability of child-friendly medications. The prevalence of prescription manipulation in Europe has been evaluated (17–19). In the UK, it was estimated that 10.1% of the prescriptions involved dose manipulations, ranging from 6.5% in regional children's hospitals to 16.3% in district general hospitals (18). A study in France indicated that approximately one-third of anti-epileptic drug prescriptions were manipulated before being administered to children (19). The intention for and practice of dose manipulation may further differ among countries that vary with regard to regulatory frameworks for drug supply and development. However, to our knowledge, the prevalence and characteristics of dosage manipulations in China have not been reported to date.

Over the past 20 years, the World Health Organization implemented legislation to encourage the development of child-friendly formulations (20). Developments and authorizations of pediatric products or supplementary information for children have primarily been promoted in the United States and Europe (21). Similar legislation and three drafts of “Wish List for Drug Development in Pediatrics” were issued in China between 2016 and 2019. However, the first product on the list was only approved in 2019, and therefore the benefits of this legislation remain limited and unknown. Considering the differences in drug supply between Europe and China, the current status of the manipulation of pediatric drugs in China remains unclear. Therefore, the aim of the present study was to estimate the dose requirement, manipulation of tablets and capsules, and inappropriate manipulations in a teaching and tertiary children's hospital in China. We further simulated the manipulation scenario based on the hypothetical supply of all drugs with reduced strength (appropriate for children) licensed by the Chinese National Medical Products Administration (CNMPA).

## Methods

We defined any drug dose manipulation as a physical alteration of a pharmaceutical drug dosage form, including the splitting, breaking, cutting, and crushing of tablets, or opening of capsules, followed by the subdivision of the dose or dispersion of only a portion of the contained liquid (10) (Supplementary Figure 1). Only manipulations made to meet an exact dose requirement were included, whereas manipulations for other purposes such as to facilitate swallowing or administration using enteral feeding tubes were not considered for this analysis. The proportion of the strength of the dose relative to that of the intact dosage form was calculated, and all prescriptions with a non-integer dose proportion value were assumed to have been provided as the exact dose followed by manipulation.

All tablets and capsules prescribed to pediatric patients (0–18 years old) during a 31-day period from March 17 to April 16, 2019, were retrieved from records of the hospital's pharmacy. The strength, which represents the amount of the drug in an intact dosage form, was obtained from the package insert. Each prescribed dose was divided by the respective strength stated on the insert to determine the proportion of dose required. All prescribed doses with a non-integer strength value, meaning that the dosage given was lower than the amount of drug in the intact dosage form, were assumed to have been administered at the exact dose, suggesting a dose manipulation (i.e., subdivision of intact drug forms carried out by splitting or crushing tablets, opening capsules, dispersing the drug in water, or by using other combined methods). Medications with the same subdivision, for example all those that were split in half, were identified as one type of manipulation. For example, splitting two medications in half were considered as one separate type of manipulation. Medications were classified according to the anatomical therapeutic code system.

Avoidable manipulation was identified as a manipulation that could have been avoided by rounding the dose up or down (within 10%) to the indicated strength, using available alternative products with the same route of administration with an appropriate strength, or using an alternative dosage form of liquid preparations with a convenient measurable volume.

Inappropriate manipulation was identified as manipulation of a medication with a narrow therapeutic index (NTI) (22), hazardous ingredients (HI) (23), or modified release (MR) dosage form (e.g., controlled release, sustained release, and modified release) unless specific information from the manufacturer permits manipulation.

Moreover, the manipulation practice was classified in terms of method (tablet splitting, tablet crushing, capsule opening, dispersion in liquid, and combined methods such as dispersion in liquid after capsule opening or tablet crushing) and provider (pharmacists, nurses, and family caregivers).

The possibility for manipulation reduction was simulated according to a hypothetical supply of all products with the lowest strength in the hospital that are approved by the CNMPA. All licensed products in China of the medications relevant to this study were retrieved from the CNMPA database (http://app1.nmpa.gov.cn/data_nmpa/face3/base.jsp?tableId=25&tableName=TABLE25&title=%B9%FA%B2%FA%D2%A9%C6%B7&bcId=152904713761213296322795806604) in September 2020, and the lowest strength of each product was recorded. If the strength of the drugs found to be subjected to manipulation during the study period was not the lowest licensed strength in China, the manipulations were re-estimated based on the lowest strength licensed by the CNMPA to determine the potential for manipulation reduction.

## Results

### Overview of Prescriptions

A total of 78,366 prescriptions (2,528 per day) of 142 medication types of any form were recorded during the 31-day period. Montelukast sodium chewable tablets 4 mg (*n* = 18,478, 24%) and 5 mg (*n* = 4,558, 6%), vitamin D drops soft capsule 400 IU (*n* = 6,081, 8%), cefdinir dispersible tablet 50 mg (*n* = 3,587, 5%), and cefdinir capsule 0.1 g (*n* = 3,054, 4%) were the most frequently prescribed medicines during the study period.

### Dose Proportion and Manipulation

**Figure 1:**
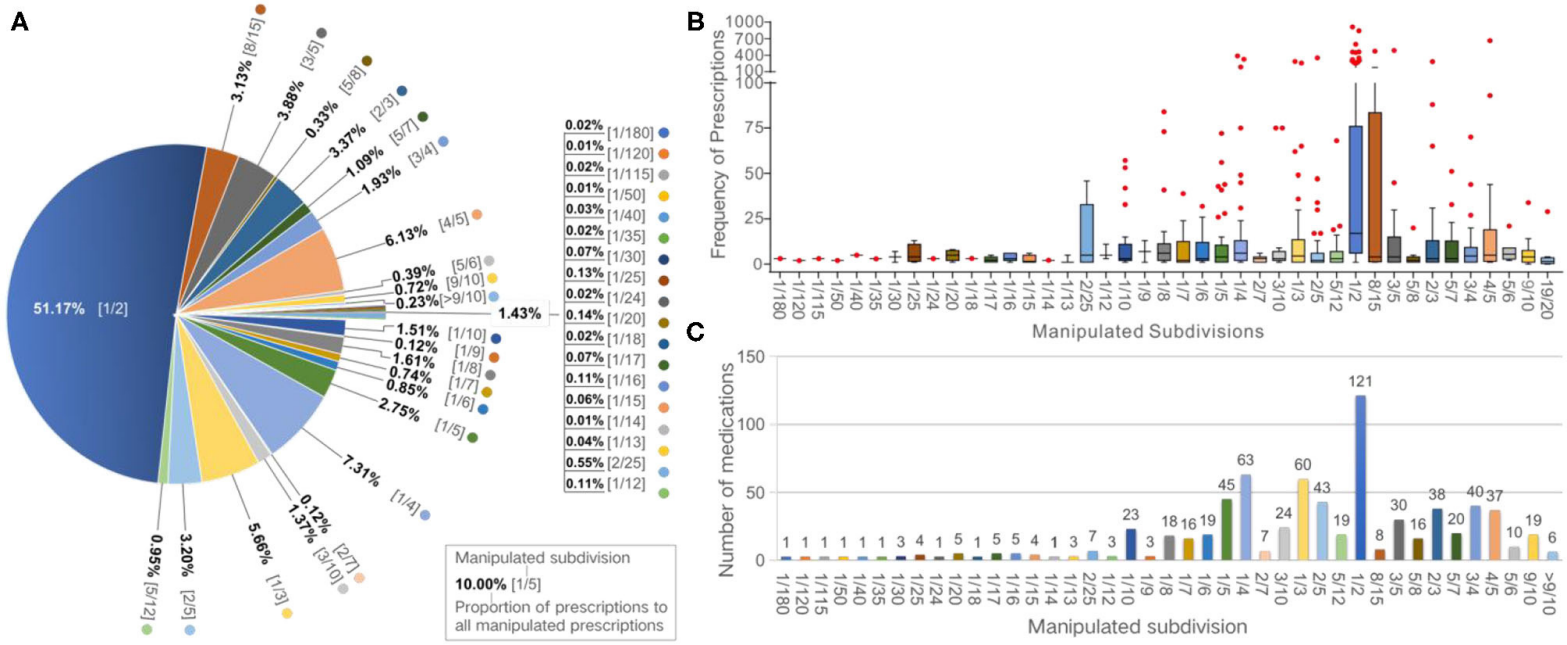
Frequencies of manipulated prescriptions and medications according to subdivisions. **(A)** Pie chart showing the proportion of manipulated prescriptions for each subdivision. **(B)** Box plot showing the frequency of manipulated prescriptions of each medication according to subdivisions. **(C)** Bar graph showing the numbers of manipulated medications according to subdivisions.

During the 1-month study period, 17,123 (22%) of the total 78,366 tablet or capsule prescriptions were assessed as requiring manipulation, comprising 109 tablets (84.5%) and 20 capsules (15.5%). Assuming all non-integral proportions of tablets/capsules were given at the exact prescribed dose, 43 different dose proportions would have been required. The top 10 common subdivisions (responding frequency, per 1,000 prescriptions), accounting for 89% of the manipulated prescriptions, were 1/2 (*n* = 112), 1/4 (16), 4/5 (13), 1/3 (12), 3/5 (9), 2/3 (7), 2/5 (7), 8/15 (7), 1/5 (6), and 3/4 (4) of the strength, which were required by 85, 44, 26, 42, 21, 27, 30, 6, 32, and 28% of all medications, and covered 51, 7, 6, 6, 4, 3, 3, 3, 3, and 2% of all manipulated prescriptions, respectively (Figure 1A). The large majority of the medicines (99.6%) and manipulation types (97.6%) were handled by family caregivers or pharmacists. Solution dispensing was only used in four types of manipulations for three medicines. Most of the manipulated prescriptions for inpatients were manipulated prior to administration by pharmacists, with a small proportion of these manipulated by nurses at the time of administration. For outpatients, most of the medicines were manipulated by the carers of the children at the time of administration, with a small proportion manipulated by pharmacists at the hospital.

### Medication

Overall, 129 of the 142 medications prescribed during the study period (90.8%) were deemed to have been manipulated. Among these, 121, 63, 60, 45, and 43 medications required subdivisions of 1/2, 1/4, 1/3, 1/5, and 2/5, respectively (Figure 1B). The median number of the manipulated prescriptions for each medication was 175 [interquartile range (IQR): 11–155; range: 1–1,749]. The median of the number of type of manipulation and the proportion of manipulated prescriptions was 4 manipulations (IQR: 2–8; range: 1–23) and 32.6% (IQR: 12.8–90.0%; range: 0.006–9.0%) per drug, respectively. Oseltamivir phosphate capsules (75 mg), levothyroxine sodium tablets (50 μg), cefdinir capsules (0.1 g), valacyclovir hydrochloride dispersible tablets (0.15 g), and cefdinir dispersible tablets (50 mg) were the five most frequently manipulated medicines, accounting for 34.7% of all manipulated prescriptions (Table 1). Moreover, all prescriptions of sildenafil tablets (25 mg), clopidogrel hydrogen sulfate tablets (75 mg), isosorbide dinitrate tablets (5 mg), amiodarone hydrochloride tablets (0.2 g), and sapropterin hydrochloride tablets (0.1 g) were manipulated.

**Table 1:** The top 10 medicines with most frequent manipulation.

**Medication and strength**	**Manipulation**				
	Orders^*^	**Types**	**Most frequent**		
			**1st^**§**^**	**2nd^**§**^**	**3rd^**§**^**
Oseltamivir phosphate capsule 75 mg	1,749 (56), 10.2	22	4/5, 34.1	3/5, 24	2/5, 16.2
Levothyroxine sodium tablet 50 μg	1,225 (40), 7.2	16	1/2, 43.4	1/4, 25.9	1/3, 15.5
Cefdinir capsule 0.1 g	1,071 (35), 6.3	7	1/2, 78.8	4/5, 8.7	3/10, 7.0
Valacyclovir hydrochloride dispersible tablet 0.15 g	1,042 (34), 6.1	12	8/15, 39.1	1/3, 20.8	2/3, 20.7
Cefdinir dispersible tablet 50 mg	867 (28), 5.1	9	1/2, 89.5	4/5, 3.9	3/5, 2.1
Nitrazepam tablet 5 mg	502 (16), 2.9	9	1/4, 51.6	1/2, 32.5	1/10, 3.0
Spironolactone tablet 20 mg	495 (16), 2.9	18	1/4, 23.6	1/2, 19.8	1/8, 14.7
Ketotifen fumarate tablet 1 mg	451 (15), 2.6	4	1/2, 84.7	1/3, 13.7	3/10, 1.3
Hydrochlorothiazide tablet 25 mg	449 (14), 2.6	20	1/8, 18.7	1/4, 16.7	1/5, 16.0
Azithromycin tablet 0.25 g	410 (13), 2.4	17	1/2, 31.2	3/5, 11.0	1/5, 10.7

* *Total manipulation orders during 31 days (per day), proportion of manipulation orders in all orders*.

§ *The subdivision with responding order proportion in all manipulation order*.

**Figure 2:**
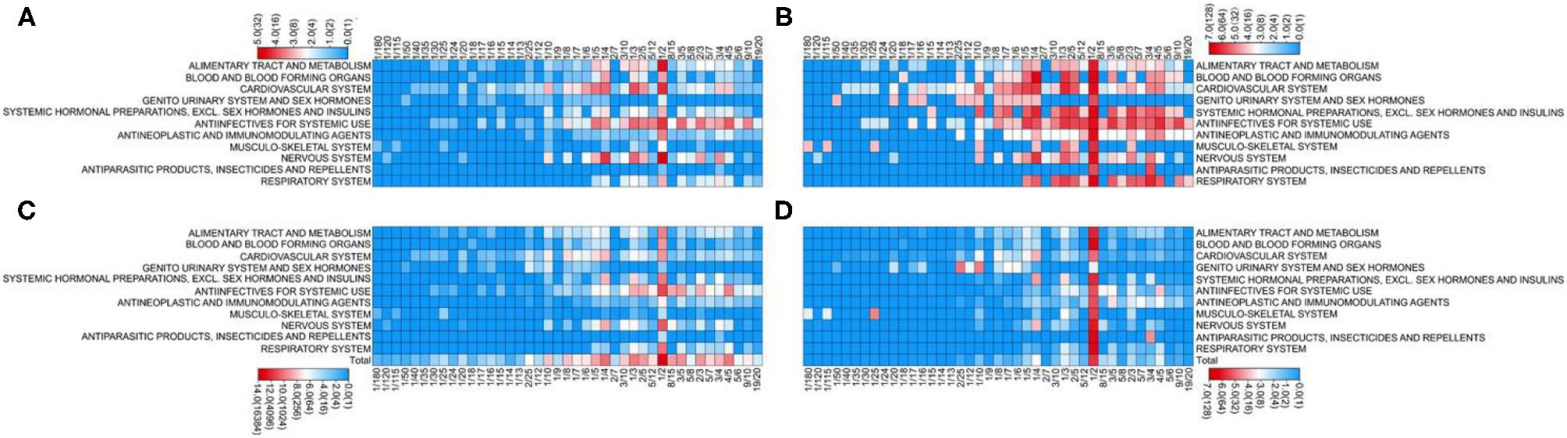
Manipulated medications and prescriptions according to the medication classification. **(A)** Number of manipulated medications. **(B)** Proportion of manipulated medications relative to all prescribed medications (%). **(C)** Number of manipulated prescriptions. **(D)** Proportion of manipulated prescriptions relative to all prescriptions (%).

The highest proportion of manipulated doses (65%) was recorded for cardiovascular system drugs, and nearly half of the medications (9/20) required manipulation for more than 75% of the doses. In comparison, the least manipulated dose proportions were observed among therapeutic agents prescribed for the respiratory system (6%), antiparasitic products, insecticides and repellents (9%), and alimentary tract and metabolism drugs (9%) (Figure 2).

### Inappropriate and Avoidable Manipulations

In total, 19.3% of the manipulated prescriptions (*n* = 3,298) were linked to medicines that were indicated to be inappropriate for manipulation. In total, 100% (11/11), 88% (22/25), and 91% (10/11) of medications with NTI, HI, or MR formulations were manipulated, accounting for 77, 39, and 13% of all prescriptions of medications with NTI, HI, or MR formulations, and 9, 8, and 3% of all manipulated prescriptions, respectively. Overall, 20.8% of the manipulated prescriptions (*n* = 3,569) could have been avoided, including 326 prescriptions (1.9%) where manipulation could have been avoided by rounding up/down the dose within a 10% error margin, 829 (4.8%) for whom it could have been avoided by changing to an available liquid oral formulation, and 2,414 (14.1%) for whom it could have been avoided by changing to an available solid oral formulation with reduced strength. In addition, 47 of the 129 manipulated medications, were manufactured at a strength lower than that available at the hospital, and this lower strength was approved by the CNMPA. If all 47 products had been available at the hospital at the required lower strength, 3,594 (21%) prescriptions comprising 46 manipulations, mostly involving halving of the tablet/capsule contents (76%), could have been avoided.

## Discussion

Manipulation of drug doses is common in the pediatric clinical setting. Approximately 10% of the doses for children require manipulation in Europe (17, 18); however, the prevalence of such manipulation at our hospital in China is double of that reported in Europe. This might be attributed to the lower number of approved age-appropriate formulations in China, and the additional limitations of drug utilization in public hospitals imposed by the local regulatory framework (24). For instance, a CNMPA-approved medication cannot be used in a public hospital unless it is authorized by the local government. Moreover, no more than two products with the same active ingredients for each administration route can be used in a public hospital, regardless of the strength or manufacturer. Our analysis indicates that if the manipulated medications could have been replaced with licensed medications of the lowest strength approved by the CNMPA, the rate of prescription manipulation could be reduced by 20% in the hospital. However, the reduction in manipulation by replacing medications with a formulation of the lowest strength is dependent on supply. The shortage of child-friendly formulations at both the national and regional levels in China has thus greatly contributed to the high incidence of manipulation observed in this study.

For example, oseltamivir phosphate capsule (Tamiflu®, 75 mg) and levothyroxine (Euthyrox®, 50 μg) were identified as the two medications with the greatest number of manipulated prescriptions in our hospital. No alternative low-strength products were available at the time in Zhejiang Province. In comparison, a powder for oseltamivir suspensions (Tamiflu®, 6 and 12 mg/mL) and low-strength levothyroxine tablets (Euthyrox®, 25 μg) were available during this period in the United States (22). If these two products had been available in our hospital, 14% of all manipulations, including 99.9% of manipulated Tamiflu® and 50.1% of manipulated Euthyrox® prescriptions, could have been avoided. Similarly, only 25 and 100-mg formulations of sildenafil are approved in China for adult erectile dysfunction. In our hospital, all related prescriptions were manipulated to obtain a 1/50 to 1/2 strength for the management of pulmonary hypertension in neonates; a powder form for both suspension (10 mg/mL) and injection (0.8 mg/mL) is currently available in Europe and the United States (22), and its availability could help avoid all of the sildenafil manipulations that occur in our hospital. Fortunately, this situation is expected to improve in China in the near future. The “Wish List for Drug Development in Pediatrics” (2016) released the first listed product, namely chloral hydrate enemas, which was approved by the CNMPA in 2019. Moreover, the regulation regarding the use of two products with the same route of administration for a single ingredient available in a public hospital has been abolished in several provinces.

In addition, a subdivision of 1/180 for indomethacin enteric-coated tablets (strength, 25 mg) was the smallest subdivision prescribed for patients during the study period. Indomethacin was prescribed for patent ductus arteriosus in premature infants with a body weight of 0.6–1 kg at a dose of 0.1–0.2 mg/kg, even though intravenous indomethacin (1 mg/mL) is recommended for this condition in the United States (25). Unfortunately, no intravenous indomethacin has been approved for use in China. Therefore, in our hospital, indomethacin is ground and dissolved in water, and a portion of the liquid is administered to premature infants.

In addition to the shortage of products, lack of awareness of related risks also likely contributes to the high incidence of manipulation. Manipulation should be avoided whenever possible to reduce potentially adverse events (8, 11, 12, 14, 15, 26–29), particularly for medicines that are not recommended for manipulation (30) such as products with NTI, HI, or MR (31). In the present study, ~20% of the documented manipulations could have been avoided by rounding the doses or choosing alternative products available in the hospital, and 20% of the recorded manipulations were linked to inadvisable manipulation. Given the current situation in China as outlined above, manipulation is more likely to be considered a routine and normal practice, and thus the doctors are not fully aware of the potential risk of manipulation. Although there is little information of the clinical impact of manipulated drugs, adverse events linked to inaccurate dose and an impaired dosage form should been considered. For products with NTI, an inaccurate dose might lead to plasma concentration levels outside the therapeutic windows. With respect to sustained-release or enteric-coated tablets, impaired dosage forms might reduce the bioavailability, shorten the time taken to reach peak concentrations, and increase peak plasma concentration levels, which can theoretically induce adverse events. Therefore, it is considered to be inadvisable to manipulate medicines containing products with NTI, HI, or MR unless such manipulation is specifically licensed by the manufacturer. Unfortunately, there is very little information regarding manipulation mentioned in package inserts. Manipulation was found to be recommended for only 4% of the tablets and capsules available in the hospital; for 6% of the medicines, it was not recommended and for another 6%, the use of an alternative product with appropriate strength or dosage forms was recommended, instead of manipulation, to meet the dose requirement of children. For the remaining 84% of the medicines, there is no information about manipulation mentioned in the insert. Therefore, most of the medicines were manipulated in the hospital without an associated recommendation of the manufacturer.

Manipulation also appears to be disease-dependent. The highest proportion of manipulated prescriptions was found for cardiovascular system drugs. Owing to the low prevalence of cardiovascular disease in children, pediatric doses of cardiovascular drugs are seldom considered in drug development. Therefore, 17 of the 20 available cardiovascular drugs prescribed during the study period were manipulated, with more than 40% of the prescriptions affected. By contrast, the lowest proportion of manipulated doses was documented for therapeutic agents against common pediatric diseases, including respiratory system drugs, anti-parasitic products, insecticides and repellents, and alimentary tract and metabolism agents. A previous study indicated that 34% of prescribed anti-epileptic drugs required manipulation in a French tertiary center for pediatric epilepsy (19), which is in line with our findings.

## Limitation

To the best of our knowledge, this is the first study to report the current status of pediatric drug manipulation in China. However, this study has several limitations. The manipulations were estimated based on the assumption that drugs that were administered exactly according to the prescribed dose, but we could not document the actual manipulation practice of health care professionals and informal caregivers of the children. We considered any non-integer dose proportion value as a manipulation. Therefore, the rate of manipulation could have been slightly overestimated, since it cannot be confirmed that all dosing proportions were actually manipulated, especially those with a prescribed dose close to the indicated product strength. In addition, we only focused on tablets and capsules in the present study, and other formulations such as sachets, nebulizer solutions, intravenous injections, suppositories, enemas, and transdermal patches were not included. Further, we only focused on prescriptions over a 1-month period, and the manipulation prevalence might vary throughout the year due to seasonal outbreaks such as those of diarrhea in the autumn and respiratory disease in the winter, as well as the increased number of surgeries over the holidays. Given that the medication with the lowest strength approved by the CNMPA may not be produced or available, the estimated 20% reduction in prescription manipulation constitutes the current ideal scenario. Finally, the clinical effect of manipulations should be explored in future studies, especially for manipulations linked to products with NTI, HI, or MR formulations.

## Conclusion

We identified a high incidence of manipulated capsules and tablets in a Chinese tertiary children's hospital. The manipulated doses may be attributed to a lack of available age-appropriate medicines, avoidable manipulation due to lack of knowledge of consequences or alternatives, and inappropriate manipulation.

## Data Availability Statement

The original contributions presented in the study are included in the article/Supplementary Material, further inquiries can be directed to the corresponding authors.

## Ethics Statement

Ethical review and approval was not required for the study on human participants in accordance with the local legislation and institutional requirements. Written informed consent from the participants' legal guardian/next of kin was not required to participate in this study in accordance with the national legislation and the institutional requirements.

## Author Contributions

LZ, CH, and LF contributed to conception and design of the study. LZ and YH collect the data. PP and CH performed the statistical analysis. LZ and YH wrote the first draft of the manuscript. LF revised the manuscript. All authors approved the submitted version.

## Funding

This work was supported by the National Natural Science Foundation of China (81773819 and 81973396), Natural Science Foundation of Zhejiang Province (Y16H160129 and LSY19H300001), and Zhejiang Provincial Program for 151 Talents (to LF).

## Conflict of Interest

The authors declare that the research was conducted in the absence of any commercial or financial relationships that could be construed as a potential conflict of interest.

## Publisher's Note

All claims expressed in this article are solely those of the authors and do not necessarily represent those of their affiliated organizations, or those of the publisher, the editors and the reviewers. Any product that may be evaluated in this article, or claim that may be made by its manufacturer, is not guaranteed or endorsed by the publisher.

## Supplementary Material

Figure S1 The procedure of manipulations.
