# Supplementary figures and images for: Estimated Manipulation of Tablets and Capsules to Meet Dose Requirements for Chinese Children: A Cross-Sectional Study
Source: Front Pediatr. 2021 Oct 29;9:747499. doi: 10.3389/fped.2021.747499 (PMC8585988; doi:10.3389/fped.2021.747499)

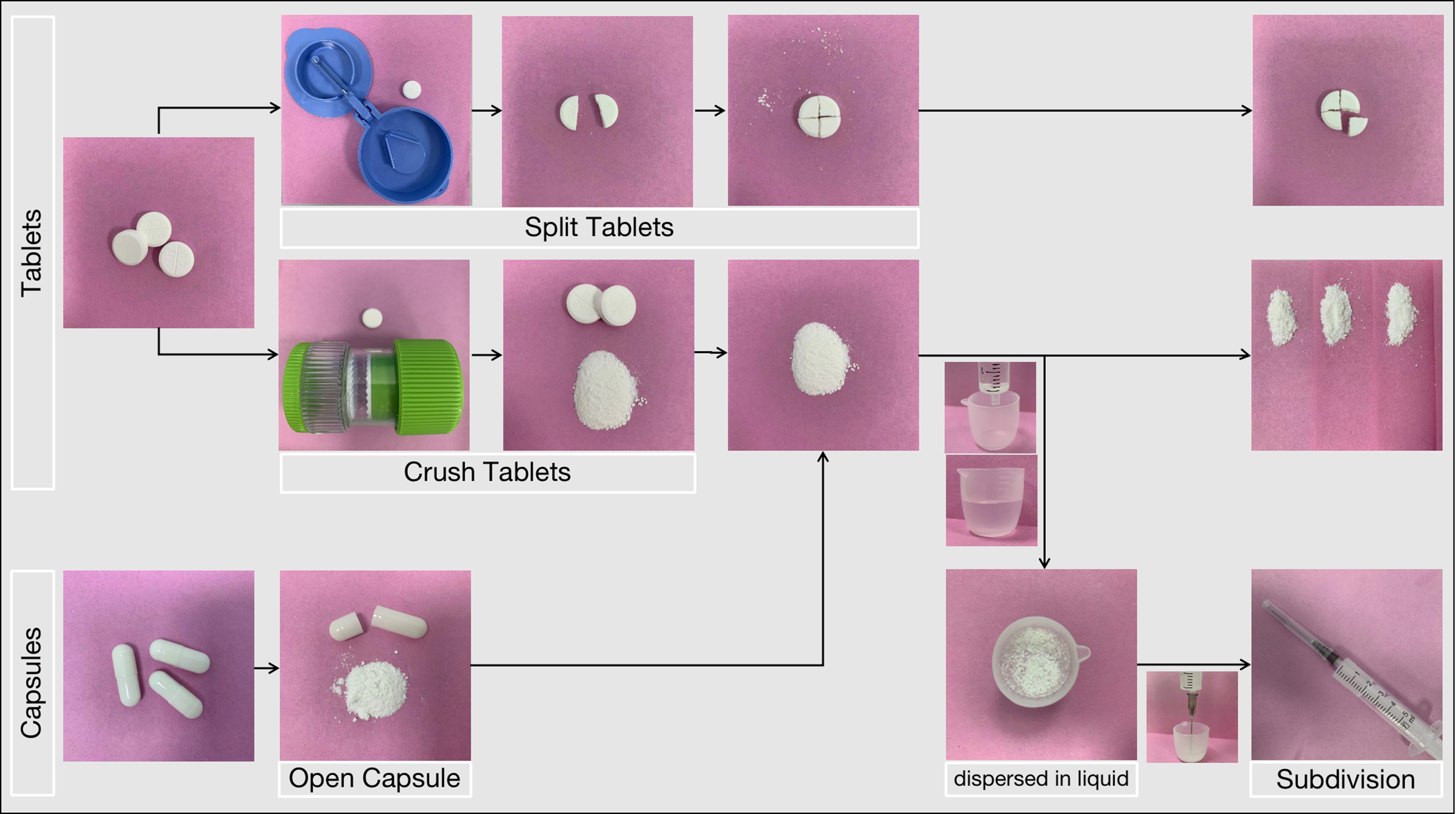

Supplement: Figure S1 — The procedure of manipulations. [file Image_1.TIF]
